# A matter of psychic life and death: reappraising the death drive and its link to psychosis through the prism of the free energy principle

**DOI:** 10.3389/fpsyg.2026.1795266

**Published:** 2026-04-14

**Authors:** Matthew John Mellor

**Affiliations:** 1 University of Exeter, Exeter, United Kingdom; 2 Essex Partnership University NHS Foundation Trust, Southend-on-Sea, United Kingdom

**Keywords:** death drive, free energy principle, Karl Friston, life drive, neuropsychoanalysis, psychosis, Sigmund Freud

## Abstract

This paper applies principles and perspectives emerging from free energy neuroscience to the psychoanalytic concept of the death drive. The aim is to offer a contemporary reappraisal of this controversial aspect of psychoanalytic theory and its link to psychosis. The paper begins with a review of the death drive as proposed by Sigmund Freud, before proceeding to briefly outline Karl Friston’s free energy principle. Building on proposals from Gustaw Sikora and Bernard Penot, it then explores how the combined and coordinating processes of minimising [binding] free energy and dismantling [unbinding] inexpedient generative models of reality may be understood as essential to life, growth, and adaptation. The question is thus raised: if a periodic unbinding—even destruction and demise—of generative models is vital to adaptive living, how might the death drive be conceptualised? The paper then proceeds to develop the notion that what Freud identified as the (defused) death drive may reflect a critical breakdown in the reciprocal ebb and flow of binding free energy/unbinding generative models of reality. Two illustrations—both of which concern psychotic phenomena—are given in an attempt to depict how the death drive in defused form may be recognised as manifesting both as arrested unbinding and/or interminable binding. The discussion explores how such a breakdown in the vital rhythms of life and self-organisation can sabotage the ability to think, compromise the mind’s capacity to function as a container, and produce a boundless infinitisation of experience therein.

## Introduction

In the wake of the First World War, Sigmund Freud produced a paper exploring psychic life *Beyond the Pleasure Principle*. In it, he introduced a concept that would become one of the most controversial aspects of his metapsychology: the death drive; a force Freud (1920) described as responsible for living organisms’ endogenous impulsion towards death. In his own words, the “*aim of all life is death*” and thus everything that lives “dies for *internal* reasons” (Freud, 1920, p. 38). Whilst a considerable proportion of the psychoanalytic community continue to embrace the concept of the death drive, the likes of Hermann (1992) argue that most theorists have rejected Freud’s ‘Manichaean’ explanation and would rigorously contest many of the proposals he advanced in 1920.

The aim of this paper is to contribute to an updating of Freud’s original theoretical model from the perspective of free energy (FE) neuroscience (c.f. Friston, 2010, 2013; Parr et al., 2022). The work orbits around an exploration of the notion that the (defused) death drive may reflect a critical breakdown in the reciprocal processes of (A) binding FE, and (B) unbinding generative models that require modification to effectively facilitate ongoing FE minimisation. Key objectives are to illustrate how this theorisation of the death drive may present and discuss its implications for understanding psychosis.

## The birth of the death drive

For the first two decades of the twentieth century, Freud posited an instinctual dualism in which self-preservative ego drives were seen to conflict with object-seeking sexual drives (Beer, 1998; Delion, 2018). Despite their potential for opposition, these libidinal energies were both understood ultimately to be dictated by the same underlying pleasure principle: the motivational logic that impels humans to seek instinctual gratification (American Psychological Association, 2023).

By 1920 however, Freud had come to view this picture as incomplete. Influenced by, amongst other things, his experience of treating soldiers returning from the trenches, many of whom reported recurrent post-traumatic nightmares, Freud began to question whether the pleasure principle alone could account for the tendency of traumatised minds and bodies to become fixated in compulsively repetitive cycles that delivered no satisfaction. What followed was a radical revision of drive theory, whereby such clinical phenomena became seen as indicating “the operation of tendencies *beyond* the pleasure principle, that is, of tendencies more primitive than it and independent of it” (Freud, 1920, p. 17).

The question was posed: “how is the predicate of being ‘instinctual’ related to the compulsion to repeat?” (Freud, 1920, p. 36). Here, for the first time, Freud (1920, pp. 36–38) introduced the notion that there exists, within organic life, an intrinsic urge *“to restore an earlier state of things”*: an “*old* state” of invariant inertia. The inherent impulsion towards such an “inorganic” state herein came to be understood as the force lying behind the “daemonic” tendency of traumatised individuals to trip endlessly around ossified psychic and somatic loops that do nothing to satisfy the pleasure principle (Freud, 1920, pp. 36–38).

Consequently, a new instinctual duality began to take shape; the old tension between self-preservative and sexual drives gave way to a more fundamental duality between life- and death drives. Having only previously conceptualised the tendency of living organisms to strive towards self-organisation, Freud thus took a turn on his intellectual journey and began to understand psychic life as liable to become impeded by a more deadly drive to enervate. Writing at the end of his life, Freud’s (1938, p. 148) final definition of this duality would take the following form:

The aim of [… Eros] is to establish ever greater unities and to preserve them thus—in short, to bind together; the aim of [… Thanatos] is, on the contrary, to undo connections and so to destroy things. In the case of the destructive instinct we may suppose that its final aim is to lead what is living into an inorganic state. For this reason we also call it the death instinct.

Despite this conceptual duality, Freud (1930, p. 121) also noted how much more conspicuous Eros is vis-à-vis Thanatos, stating that we can only suspect the latter “as something in the background behind Eros” which mostly escapes detection unless revealed by being “alloyed with Eros.” However, as Laplanche and Pontalis (1973, p. 100–101) note, in certain circumstances it may be possible to glimpse the death drive in its “pure state […] defused from the life instinct, as in the case of the melancholic.” Such defusions and their potential relevance to the psychoses will move into focus as this discussion progresses.

## A contemporary reappraisal

From today’s vantage point, following a century of scientific advancement, there remains keen debate over the validity of these ideas. A significant development during recent years has been the emergence of the *free energy principle* (FEP). Pioneered by Karl Friston, FEP represents a paradigmatic shift within the neurosciences that, for Holmes (2022, p. 164), offers psychoanalysis “new life “due to its contemporaneity and compatibility with psychoanalytic thinking. Before Fristonian FEP is applied to considering the death drive, a brief overview is necessary.

### Friston’s free energy principle

FEP proceeds from the fact that “we live in an entropic universe. […] Coffee cools once poured. Stars burn out” (Holmes, 2022, p. 165). However, amid this “cosmic tide towards disorder and homogeneity,” there exist pockets of resistance: “life itself” (Holmes, 2022, p. 165). For organisms to be alive, they must necessarily engage in what Erwin Schrödinger termed ‘negentropy’ (negative-entropy) (Holmes, 2022, p. 165). For Friston (2010, p. 127), this natural tendency to resist entropic disorder is intimately linked with the ability to minimise “informational “surprise””; that is, “the negative log-probability of an outcome, i.e., how ‘likely’ or ‘unlikely’ a particular event, from a specific organism’s viewpoint,” is to occur (Holmes and Nolte, 2019, pp. 1–2).

In the FEP framework, organisms are understood to minimise surprise by generating “predictions” about the sensations they experience within their given environment (Friston, 2010, pp. 127–128). Thus, if an event is “probable to a high degree,” the surprise is minimal and “little new information is gained” (Holmes and Nolte, 2019, p. 2). By contrast, improbable events that produce high levels of informational surprise are experienced as such due to failures of and/or “*errors*” within the organism’s predictive model (Friston, 2010, p. 130, my italics). Advocates of FEP argue that predictive processing of this order is what enables organisms—from microbes to men (albeit with widely varying levels of sophistication)—to sense when they have departed from the limited range of internal states required to sustain homeostasis (Holmes, 2022).

When predictions do not match reality, the “surprise” [corresponding to “prediction error” (Friston, 2010, p. 130)] generated means that the organism in question will experience a biological demand to restore their internal equilibrium (Sikora, 2022). As Friston (2010) and colleagues (e.g., Parr et al., 2022) have shown, the degree of prevailing surprise can be measured by calculating the quantity of FE present within the organic system. It is worth noting here that this description of FE corresponds closely to how neuropsychoanalytic figures such as Solms (2021, p. 96) have conceptualised the function of affectivity: that is, as a signal for “*how you are doing* in relation to your biological needs.”

By organisms employing active (Bayesian) inference—and therein developing, testing, and updating the generative models with which they perceive the world to better fit the sensory data—they enhance their potential to minimise FE (Holmes, 2022; p. 166; Friston, 2010; Mellor, 2018; Joyce, 2008). In addition to such modifications of perception, acting on the world to address the causes of informational surprise offers another route to binding manifest FE (Friston, 2010, p. 128). Crucially, without living organisms evincing these properties amid an external world that is forever in flux, their capacity for negentropy is jeopardised and their “life as we know it” renounced (Friston, 2013, p. 1).

### Sikora’s application of the free energy principle

In his article on *The Drives from Friston’s Free Energy Perspectiv*e, Sikora (2022) maps FEP directly onto Freud’s (1915, p. 121–122) concept of the “instinct [drive]”: “the psychical representative […] of the demand made upon the mind for work.” Sikora (2022, p. 2) thus proposes that FE:

can be seen as a biophysical source of the drives, and that the process of minimizing this free energy is equivalent to what Freud described as a process of binding energy […] It offers an answer to the question of what force is behind crucial processes of cathexis/de-cathexis.

Contextualising his thesis by drawing on André Green, Sikora (2022, p. 2) proposes an understanding of the drives that regards them as “forces of binding and unbinding (investment and disinvestment, cathexis/de-cathexis).” However, unlike Green who, as Sikora (2022, p. 5) argues, “sees the energy of the death drive as a force of dis-objectification/unbinding,” the death drive is not, for Sikora, exclusively linked with unbinding. Following Penot (2017), he instead contends that only the coordinating (or, as it were, “alloyed”) coexistence of binding *and* unbinding can promote change, growth, and life: “without de-cathexis, there will be stagnation and no development will be possible. Without cathexis, there will be chaos.” (Sikora, 2022, p. 8).

Consequently, both cathexis and de-cathexis—understood here as corresponding to the mutual processes of minimising (binding) FE and dismantling (unbinding) generative models that require adjustment—are understood as key to life and adaptation. Without both, the psyche cannot establish the requisite balance between “stability” and “room for change” that FEP shows is vital for negentropically maintaining homeostasis (Sikora, 2022, p. 5).

The question then follows: how might a more “defused” expression of the death drive be conceptualised? Crucially for Sikora (2022, p. 13), the death drive constitutes not so much a “sole force,” but rather *a breakdown in the rhythmic ebb and flow of binding/unbinding processes*. The result of such a breakdown is said to be a “severe imbalance” between the operation of the two dialectical forces (Sikora, 2022, p. 13). Returning to Freud’s trauma-informed theory, it is precisely “too much surprise (free energy)” that Sikora (2022, p. 13) argues can induce this imbalance by overwhelming the mutual functioning of the drives and disrupting the development this facilitates.

This perspective raises a possibility: could what has been termed the death drive express differently depending on how, across the binding-unbinding spectrum, the weight of such an imbalance is distributed? If so, might the (defused) death drive manifest both as *arrested unbinding* and/or *interminable binding*?

## Illustrations

Examples concerning individuals experiencing psychosis will be given. The relevance of the death drive to psychotic disorders is emphasised by the authors of *The New Dictionary of Kleinian Thought,* for whom “there is considerable agreement of there being a destructive anti-life or life-hating force that […] can induce psychosis” (Bott Spillius et al., 2011, p. 302). Moreover, the notion that free energetic overload in trauma can result in the breakdown of processes that might otherwise support psychic life and potentially lead to severe mental illness is lent empirical support by recent systematic review findings on *The Role of Childhood Trauma in Psychosis and Schizophrenia* (Inyang et al., 2022, p. 10); as the authors note “neurobiological processes occur in the brain after trauma, predisposing individuals to developing psychosis.” The following two brief illustrations depict what may be understood as the subjective and psychotic reality of: (1) arrested unbinding; (2) interminable binding.

### Illustration 1: arrested unbinding

Saks’ (2007) autobiography about living with schizophrenia—*The Centre Cannot Hold—*offers a powerful glimpse into the experience of psychotic fragmentation. Recounting a childhood memory of having disappointed her father, Saks (2007, pp. 12/13) writes:

And then something odd happens: My awareness grows fuzzy […] I think I’m dissolving […] like a sandcastle with all the sand sliding away in the receding surf. This is scary, please let it be over! Most people know what it’s like to be seriously afraid […] ‘disorganisation’ is a different matter altogether […] One’s centre gives way.

Saks’ account shows, with visceral clarity, the experience of a mind that “delinks, fragments, and unbinds” (Reed and Baudry, 2005, p. 132). Within the model explored here, such mental activity would be regarded as the result of a psyche that is unable right itself, reorganise, and bind emergent FE. The terrifying subjective consequence, as Saks evocatively illustrates, would be to feel one’s endostructure swept away in an entropic slide “towards disorder and homogeneity” (Holmes, 2022, p. 165).

### Illustration 2: interminable binding

Freud’s (1911, pp. 62–78) Schreber case offered psychoanalysis one of its first insights into paranoia and dementia praecox (or, as Eugen Bleuler would term it, ‘schizophrenia’). In it, Freud (1911, pp. 14–19) describes Schreber as presenting in a manner frequently witnessed in the psychoses wherein causal connections are seen and felt to exist between all things, including between the self and “God Almighty”:

His delusional ideas gradually assumed a mystical and religious character; he was in direct communication with God, he was the plaything of devils, he saw “miraculous apparitions”, he heard “holy music”.

Viewed through the prism of the arguments put forward, an individual apprehending links that span the cosmos and converge on the self in this way might be regarded as the product of a mind stuck in a state of relentless binding. For Schreber, as for other individuals with “messianic psychotic” (Perez, 1977) beliefs, the very thing preventing the emergence of “a sense of reality” (Johns, 2019) may in fact be the psyche’s defused incapacity to delink a proliferating web that binds without boundary.

## Discussion

The reality of psychosis is that the diverse states illustrated above might, at different moments, emerge within the same mind. This has long been recognised, including by early pioneers of psychoanalysis. Abraham (1911, pp. 141–148) for instance—one of the first to work psychoanalytically with psychotic patients and analyst of Melanie Klein herself—observed in his *Investigation and Treatment of Manic-Depressive Insanity* the frequent recurrence of “depressive” phases that can produce feelings of “disappearing from the world without leaving a trace”; a description that resonates with Illustration 1. By contrast, the “frenzy of freedom” and “grandiose ideas” that characterise the “manic phase” were, like in Illustration 2, noted by Abraham (1911, pp. 142–151) to often “attain enormous proportions”, potentially placing the individual at the centre of a world-spanning web of influence.

Whilst these brief illustrations do not capture the myriad forms that psychosis can and does take, a consistent feature of the illness—particularly during acute episodes—arguably is that subjective reality becomes liable to erupt into “divergent” infinity (c.f. Illustration 1), and/or “convergent” infinity (c.f. Illustration 2), to draw on the language of Ignacio Matte Blanco (Priel, 1994). What prevails during such moments may thus be seen not only as a stunning of the reciprocal rhythms of binding/unbinding, but also as a literal and profound lack of *intrapersonal “containment”* (Bion, 1970, p. 80, my italics); in both illustrations, the mind is no longer felt to be demarcated by the boundaries that might otherwise encompass it. To explicitly integrate a Fristonian dimension to the description, psychotic breakdowns in the mind’s capacity to function as a container—and the infinitisation of experience that can proliferate therein—may, I am suggesting, reflect a critical breakdown in the cyclical processes of binding FE by deploying predictions that actively infer the causes of surprise, and unbinding generative models that are inexpedient for processing the prevailing sensory information [essential, in Bion (1962) terms, to *learning from experience*].

Whilst a criticism of this proposal may be that it merely reaffirms, within a new lexicon, well-established theoretical constructs in a way that is not obviously enriching, I would argue that by introducing a Fristonian frame of reference, we stand to sharpen our perspective. Indeed, if we take seriously the notion that in psychotic illness there is “a destructive anti-life […] force that has the upper hand” (Bott Spillius et al., 2011, p. 302), incorporating FEP facilitates an elaboration of the life force at risk of foreclosure; that is, the actively inferential capacity to develop, test, and update predictive models of reality that carry a functional degree of probability-weighted precision and complexity. To be clear, this conceptualisation regards active inference as constituting a “thick description” (Geertz, 1973, p. 3) of the life-supporting processes involved in *thinking* (at both more and less conscious levels about homeostasis-disrupting affectivity roused in response to an ever-changing world). In this respect, it builds on Lombardi (2016, p. 50, my italics) observation that “it is exactly the *not-thinking* that causes [the patient] to fall into the trap of insanity.”

For clinicians treating those in the grip of psychosis, working to develop active inference may thus be understood as central to the therapeutic task. As many from the post-Kleinian/Bionian tradition of psychoanalysis have shown through work with psychotic individuals (c.f. Bion, 1957; Segal, 1957; Rosenfeld, 1965; Hinshelwood, 2004; Lombardi, 2016, etc.), an essential part of this task involves enabling the patient to lean on their therapist’s capacity to provide interpersonal containment via the reverie-based processing and interpretation of that which is *not-thought* [i.e., “unmentalized” (Debbané et al., 2016, p. 13)]. To work effectively in this way is, by extension, to potentially attenuate the disorganising expressions of the defused death drive, a phenomenon explored here in terms of its antagonistic repercussions for the capacity to move flexibly between binding and unbinding functions.

## Conclusion

Rather than constituting a singular instinctual imperative, the clinical manifestations conventionally attributed to the death drive have been understood as being produced by a breakdown in the vital rhythms of life and self-organisation. This paper has argued that these rhythms and the processes that constitute them may be described with a heighted degree of precision by integrating the conceptual apparatus of FE neuroscience. Indeed if, as the Grateful Dead’s Mickey Hart observed, “life is about rhythm […] we are a rhythm machine,” then breakdowns of this order—instantiated by traumatic and free energetic overload—stand to potentially compromise the very essence of what it means to be psychically alive (van der Kolk, 2015, p. 74). It is in this sense that Freud, through his articulation of the death drive and his recognition of the psychopathological significance of its defusion, may have intuited with considerable accuracy how internal worlds can become frozen within life-depleting grooves wherein *not-thinking* may be felt to offer the “least-bad” (Solms, 2018, p. 9) solution.

## Data Availability

The original contributions presented in the study are included in the article/supplementary material, further inquiries can be directed to the corresponding author.
